# Active regulator of SIRT1 is required for cancer cell survival but not for SIRT1 activity

**DOI:** 10.1098/rsob.130130

**Published:** 2013-11

**Authors:** John R. P. Knight, Simon J. Allison, Jo Milner

**Affiliations:** Department of Biology, University of York, York YO10 5DD, UK

**Keywords:** active regulator of SIRT1, regulation of SIRT1, p53 acetylation

## Abstract

The NAD^+^-dependent deacetylase SIRT1 is involved in diverse cellular processes, and has also been linked with multiple disease states. Among these, SIRT1 expression negatively correlates with cancer survival in both laboratory and clinical studies. Active regulator of SIRT1 (AROS) was the first reported post-transcriptional regulator of SIRT1 activity, enhancing SIRT1-mediated deacetylation and downregulation of the SIRT1 target p53. However, little is known regarding the role of AROS in regulation of SIRT1 during disease. Here, we report the cellular and molecular effects of RNAi-mediated AROS suppression, comparing this with the role of SIRT1 in a panel of human cell lines of both cancerous and non-cancerous origins. Unexpectedly, AROS is found to vary in its modulation of p53 acetylation according to cell context. AROS suppresses p53 acetylation only following the application of cell damaging stress, whereas SIRT1 suppresses p53 under all conditions analysed. This supplements the original characterization of AROS but indicates that SIRT1 activity can persist following suppression of AROS. We also demonstrate that knockdown of AROS induces apoptosis in three cancer cell lines, independent of p53 activation. Importantly, AROS is not required for the viability of three non-cancer cell lines indicating a putative role for AROS in specifically promoting cancer cell survival.

## 2. Background

SIRT1 is the human homologue of the Sir2 deacetylase and has been studied widely as a factor in human disease. Functionally, SIRT1 is able to regulate cellular metabolism, epigenetic gene expression and multiple target proteins important to the cellular response to stress [1]. Misregulation of SIRT1 is implicated biochemically and genetically in diabetes and has been proposed as a therapeutic target in neurodegeneration, osteoarthritis and cardiovascular disease [2–8]. SIRT1 also has a role in tumour suppression in non-transformed cells via maintenance of genomic stability [9–11]. However, cellular and molecular studies have revealed a pleiotropic role for SIRT1 in cancer, as it acts as a tumour promoter post-cancer formation [12,13].

Cellular analyses have implicated SIRT1 in tumour cell growth, and poor prognosis in hepatocellular carcinoma [14–16]. Others have reported SIRT1 overexpression in primary and murine studies of prostate cancer [17,18] and found a requirement for SIRT1 for pro-survival signalling in oestrogen positive breast cancer, where expression also correlates with poor prognosis [19,20]. SIRT1 is also over-expressed and required for cell survival in pancreatic cancers [21,22], has been implicated in the non-solid tumour chronic myelogenous leukaemia [23,24] and suggested as an anti-cancer target for medulloblastoma [25,26].

At the molecular level, SIRT1 promotes tumour cell survival via inactivation of key tumour suppressor proteins such as the transcription factors p53 [27,28] and the FOXOs [29,30], the transcriptional repressor pRb [31] and the signalling phosphatase PTEN [32]. Acetylation of p53 at a number of residues, including the SIRT1 target site lysine 382 (lysine 379 in the mouse), is essential for transactivation of genes required for cell cycle arrest and the induction of apoptosis [33]. Activation of these genes forms the p53-mediated stress response and plays an important part in the role of p53 as a tumour suppressor. As such, reduction of p53 acetylation by SIRT1 will contribute to reduced tumour suppression via p53. Additionally, SIRT1 promotes the tumorigenic functions of the proto-oncogenes c-Myc and N-Myc by modulating transcriptional co-activation, phosphorylation and protein stability [34–37]. Furthermore, SIRT1 has been linked to modulation of cancer epigenetics via regulation of histone acetylation [38], and may be enhanced by the altered metabolic status of many cancer cells via its dependence upon NAD^+^ as a coenzyme [39].

The regulation of SIRT1 also supports a role in tumour growth and survival [40]. For example, N-Myc and c-Myc complete positive feedback loops that promote SIRT1 expression and activity [35,36], and the negative regulator of SIRT1 hypermethylated in cancer 1 (HIC1) is commonly epigenetically repressed in cancer, allowing for an increase in SIRT1 activity [41].

This study focuses on protein-level regulation of SIRT1 by active regulator of SIRT1 (AROS; also known as RPS19BP1) [42]. Despite being the first reported post-translational regulator of SIRT1, little is known regarding the role of AROS in the context of SIRT1-related disease. AROS directly binds at a site (amino acids 114–217) distal to the SIRT1 catalytic domain (amino acids 245–495), and via this interaction promotes SIRT1 deacetylation activity [42,43]. AROS function has been correlated with SIRT1 in regulation of the heat shock transcription factor 1 (HSF1) [44,45]. AROS suppressed HSF1 acetylation and increased its promoter occupancy and target transcription, correlating with the role of SIRT1. However, it is not known whether SIRT1 *requires* AROS for all activity; although AROS has been reported to promote SIRT1 activity, it is possible that SIRT1 functions in the absence of AROS in certain cellular contexts. In reconstituted *in vitro* SIRT1 deacetylase assays using purified factors, SIRT1 is able to deacetylate p53 in the absence of AROS [46,47]. However, the contribution of AROS to SIRT1 deacetylase activity in a more physiological setting has not been studied in detail.

Supplementary to regulation of SIRT1, AROS also directly associates with the ribosomal protein RPS19 [48]. Through this interaction, AROS promotes the assembly of small ribosomal subunits, with depletion of the protein perturbing ribosome biogenesis and limiting global protein synthesis [49]. Interestingly, AROS may also regulate protein synthesis via a continued association with mature small ribosomal subunits and the suppression of eIF2α phosphorylation at serine 51, a key limiting step in the initiation of protein synthesis [49].

The initial characterization of AROS as a regulator of SIRT1 found that AROS depletion reduced the viability of the HCT116 cancer cell line [42]. Conversely, the viability of HEK293 cells, of non-cancerous origin, was not affected by knockdown of AROS [45]. This is consistent with AROS cooperating with SIRT1 to determine cell fate—we previously identified SIRT1 as a survival factor for epithelial cancer cell lines, whereas SIRT1 was redundant for survival of cell lines of non-cancerous origin [50]. Importantly, the role of AROS in regulating both cancer and non-cancer cell fate has not been addressed by a dedicated study. We address this here by using cell lines of both cancerous and non-cancerous origin to explore the role of AROS in cell viability.

## 3. Results and discussion

### 3.1. Active regulator of SIRT1 expression does not correlate with SIRT1

**Figure 1.**
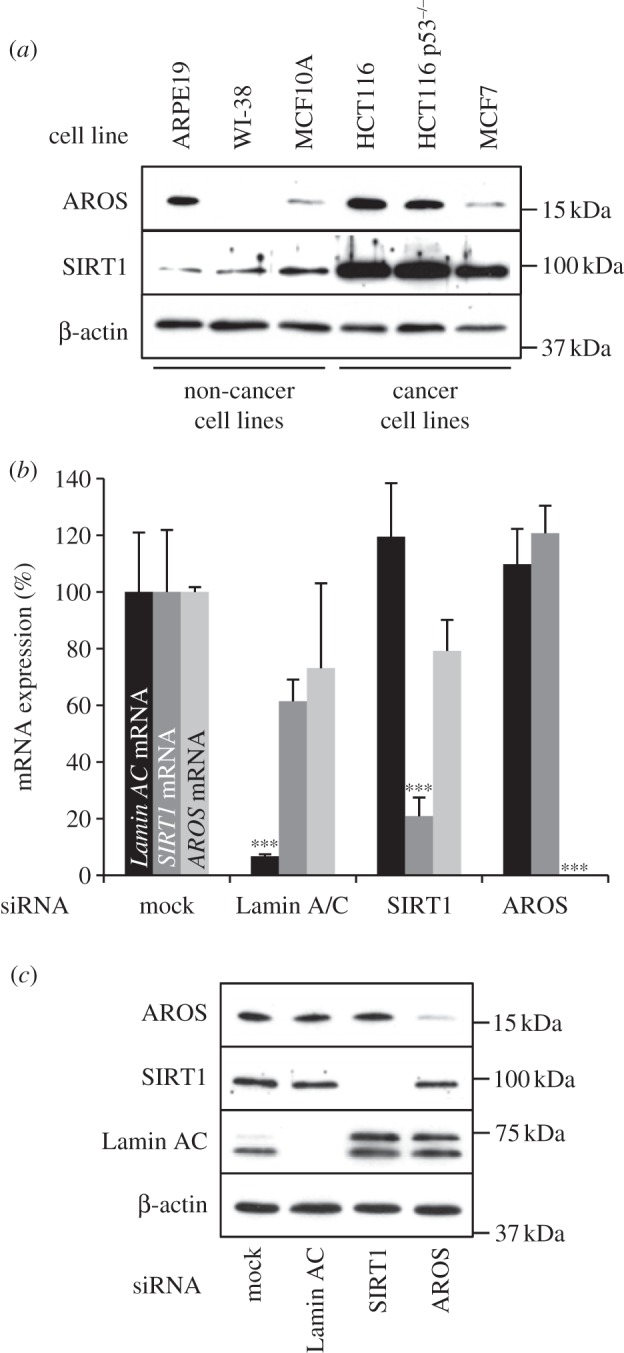
AROS expression in cancer and non-cancer cell lines. (*a*) Western blotting for the expression of AROS and SIRT1 proteins across a panel of cancer and non-cancer cell lines (see Materials and methods). β-actin is used as a loading control. (*b*) Quantification of *Lamin AC*, *SIRT1* and *AROS* mRNA by qRT-PCR from total RNA extracted from ARPE19 non-cancer cells 72 h post-transfection. ***p < 0.001. (*c*) Western blotting for expression of proteins targeted by RNAi in ARPE19 cells in parallel to (*b*). Equal loading of protein is assessed using β-actin.

We hypothesized that similar expression patterns of SIRT1 and AROS would suggest a consistent regulatory relationship between the two proteins. To assess this, we compared the protein expression of SIRT1 and AROS across a panel of human cell lines by western blot (figure 1*a*). AROS shows variable expression across the panel, and does not correlate with SIRT1 expression. Taking densitometry readings and plotting values for SIRT1 against AROS for each cell line gives a *R*^2^-value of 0.394, indicating this poor correlation (see electronic supplementary material, table S1). Taking an example, AROS expression is comparable between the MCF10A and MCF7 cell lines, whereas the expression of SIRT1 is more than twice as great in the MCF7 line. These expression patterns imply a variance in the SIRT1/AROS relationship. We also note the higher expression of SIRT1 in the cancer cell lines compared with the non-cancer cell lines (figure 1*a*).

We proceeded to analyse the function of AROS, using SIRT1 function for comparison. To do this, both *SIRT1* and *AROS* were targeted by RNAi in parallel to knockdown of a positive control mRNA, *Lamin AC*. RNAi against *AROS* reduces its mRNA expression, such that it is not detectable by qPCR in ARPE19 cells (figure 1*b*) and leads to depletion of AROS protein (figure 1*c*). Both *SIRT1* and *Lamin AC* mRNA and protein are also effectively and specifically reduced by siRNA transfection with the relevant siRNAs (figure 1*b,c*).

### 3.2. Active regulator of SIRT1 promotes cancer cell line survival

**Figure 2.**
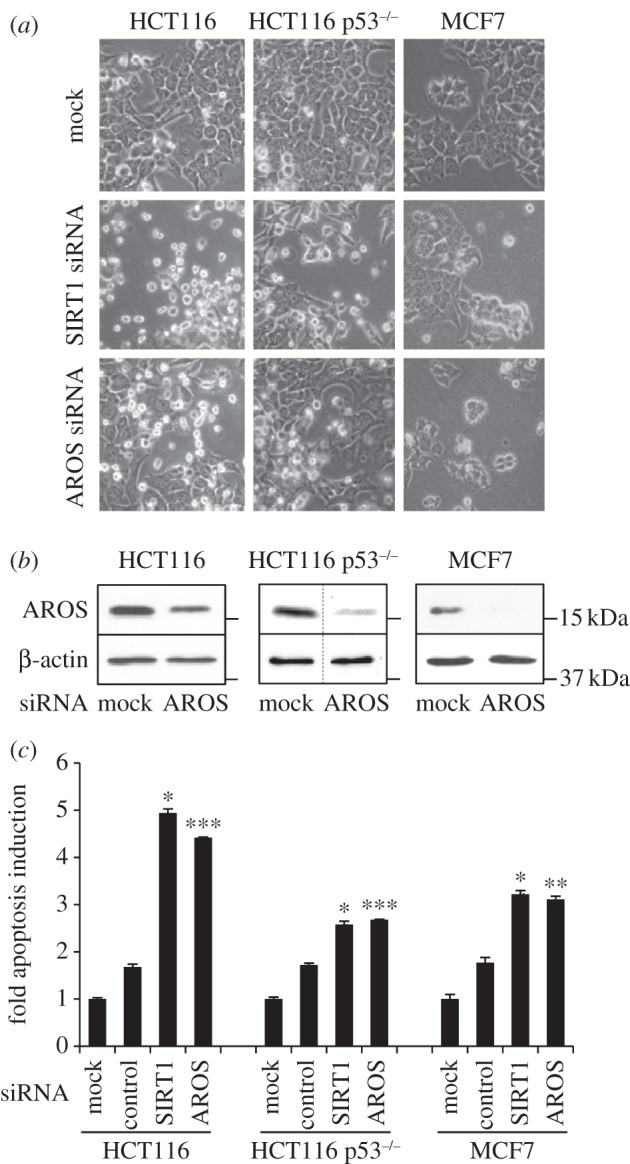
AROS promotes cancer cell line survival. (*a*) Phase contrast micrographs 48 h following targeting of AROS or SIRT1 in HCT116, p53 wild-type and null (p53^−/−^), and MCF7 cancer cells. (*b*) Western blotting for AROS protein following RNAi in each cell line with β-actin used as a loading control. Dashed lines within blots indicate removal of lanes from original autoradiographs. (*c*) Quantification of apoptotic induction in each cancer cell line following silencing of SIRT1 or AROS, including a ‘control siRNA’ that does not induce apoptosis (Lamin AC siRNA in HCT116 isogenic lines; LDH-B siRNA in MCF7 cells). Values are expressed relative to mock transfection, set to 1.0. ***p < 0.001, ** *p* < 0.01.

**Figure 3.**
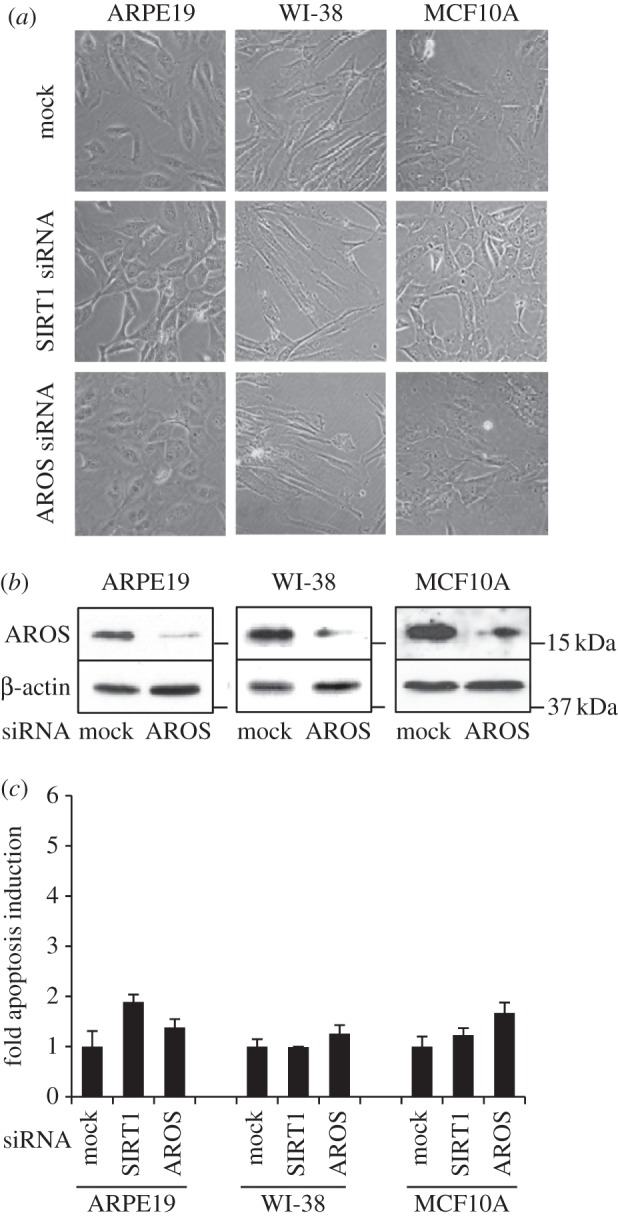
AROS is redundant for survival in non-cancer cell lines. (*a*) Phase contrast micrographs 72 h following targeting of AROS or SIRT1 in ARPE19, WI-38 and MCF10A non-cancer cells. (*b*) Western blotting for AROS protein following RNAi in each cell line. β-Actin is used as a loading control. (*c*) Quantification of apoptotic induction following targeting of SIRT1 and AROS in non-cancer cells. Apoptosis following mock transfection is set to 1.0.

We next analysed the roles of SIRT1 and AROS in cell line viability, using RNAi in a panel of cell lines with defined origins (figures 2 and 3). Suppression of either SIRT1 or AROS in three epithelial cancer cell lines results in an increase in refringent cells, consistent with an induction of apoptosis, along with a concomitant reduction in the adhered cell population (figure 2*a*). Effective knockdown of AROS is confirmed by western blotting in each cell line (figure 2*b*). The apoptotic phenotype was quantified by flow cytometry for annexin V positive and propidium iodide negative cells, which are significantly induced following silencing of either SIRT1 or AROS in each of the cancer cell lines (figure 2*c*). AROS knockdown induces a 4.4-fold increase in apoptosis in the HCT116 cell line compared with mock-treated cells, with 2.7-fold and 3.1-fold induction seen in the HCT116 p53^–/–^ and MCF7 lines, respectively. These values are comparable with the apoptotic induction following silencing of SIRT1 in each cell line (figure 2*c*). Co-knockdown of AROS and SIRT1 in HCT116 cells results in a similar induction of apoptosis to knockdown of each individually (see electronic supplementary material, figure S1*a*). This non-additive induction of apoptosis could imply that AROS suppresses apoptosis via its activation of SIRT1—loss of AROS cannot further reduce RNAi-depleted SIRT1 activity. However, it is possible that single knockdown of each target induces maximal apoptosis, and as such additive apoptosis upon co-knockdown was not registered, or alternatively that knockdown of each target converges on this induction of apoptosis via different routes.

Importantly, in each of the cancer cell lines targeting of a control mRNA does not induce an increase in apoptosis, indicating that AROS or SIRT1 suppression-induced apoptosis was not the consequence of functional RNAi (figure 2*c*). An independent siRNA, AROS 2 siRNA, induces a similar alteration in phenotype in the HCT116 cells to the first AROS siRNA (see electronic supplementary material, figure S1*b*). A significant 2.3-fold increase in apoptosis is seen following AROS 2 siRNA treatment, supporting the role for AROS in HCT116 cancer cell line survival.

### 3.3. Active regulator of SIRT1 is redundant for non-cancer cell line survival

The observation that AROS silencing induces apoptosis in three cancer cell lines is consistent with SIRT1 requiring AROS to promote survival. This led us to analyse the role of AROS in non-cancer cells, where SIRT1 is not essential for survival [50]. Suppression of either SIRT1 or AROS in ARPE19, WI-38 and MCF10A cell lines does not greatly alter cell phenotype compared with mock treatment (figure 3*a*). AROS suppression by RNAi is comparable with the level seen in the panel of cancer cell lines (figures 3*b* and 2*b*). Despite the efficient knockdown, silencing of either SIRT1 or AROS does not increase the number of apoptotic cells, measured by flow cytometry, compared with mock treatment in each of the non-cancerous cell lines (figure 3*c*).

These data indicate that AROS is required for the survival of three human cancer lines, but is redundant for viability in three cell lines of non-cancerous origins. Furthermore, AROS is able to promote cancer cell survival in the absence of p53 expression, demonstrated by apoptotic induction in the HCT116 p53^–/−^ line (figure 2*c*). Apoptotic induction is lower in these p53 null cells compared with wild-type cells, perhaps indicating that p53 facilitates apoptosis when expressed. Importantly, the data for AROS correlate with the role of SIRT1 in all cell lines analysed (figures 2 and 3 and [50]). These data also suggest that targeting AROS in cancer may be of therapeutic benefit.

### 3.4. Active regulator of SIRT1 does not influence SIRT1 expression

**Figure 4.**
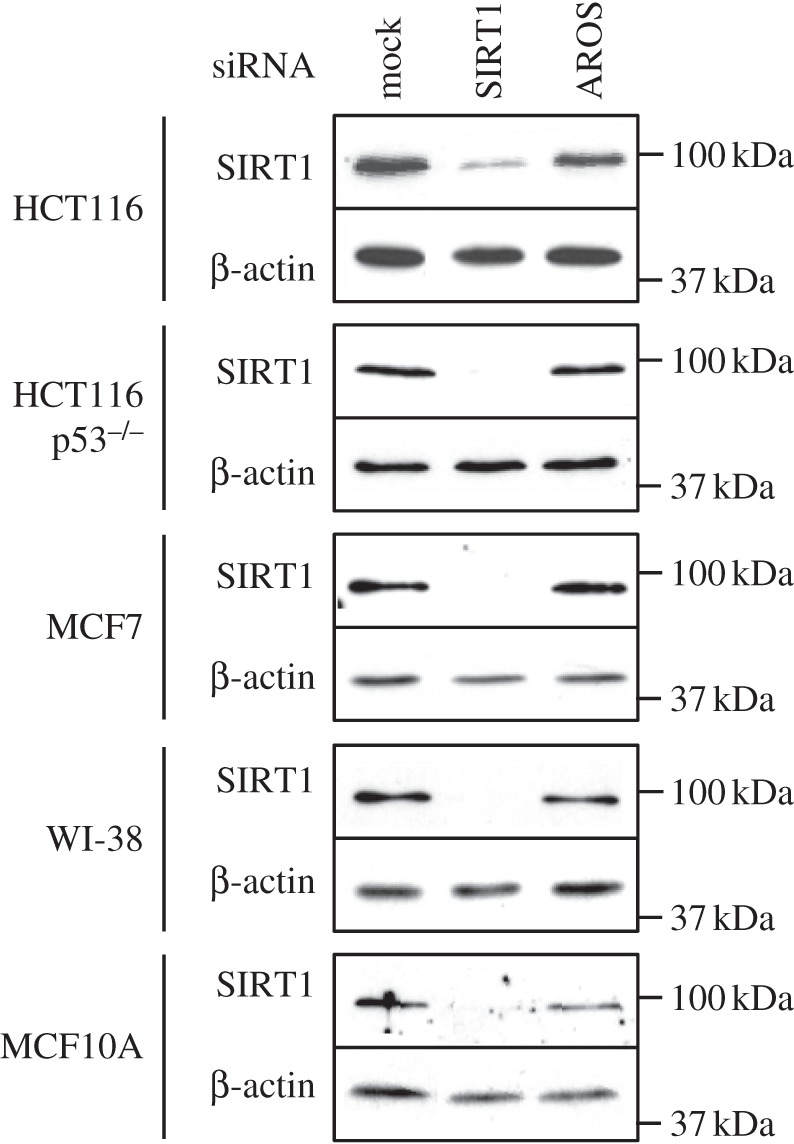
AROS knockdown does not affect SIRT1 protein abundance. Western blotting for SIRT1 protein following RNAi against SIRT1 and AROS in the panel of cancer and non-cancer cell lines. β-Actin expression is used as a loading control.

The similarity in phenotype following AROS or SIRT1 knockdown suggests similar function, and is consistent with SIRT1 activity being promoted by association with AROS [42]. Similar to the original report of AROS function, knockdown of AROS does not alter SIRT1 protein expression in the panel of cell lines analysed (figure 1*c* and figure 4), aside from a slight decrease in SIRT1 expression in the MCF10A cell line following AROS knockdown. AROS knockdown does not negatively affect *SIRT1* mRNA expression in the ARPE19 cell line (figure 1) or the other five cell lines analysed (see electronic supplementary material, figure S2). Figure 4 illustrates SIRT1 silencing resulting in the phenotypes in figures 2 and 3. To this point, the data presented correlate with a role for AROS in promoting SIRT1 function at the cellular level, despite differences in relative expression of the proteins between cell lines. We next analysed the role of AROS in the molecular function of SIRT1-deacetylation.

### 3.5. Active regulator of SIRT1 suppression of p53 acetylation is variable

**Figure 5.**
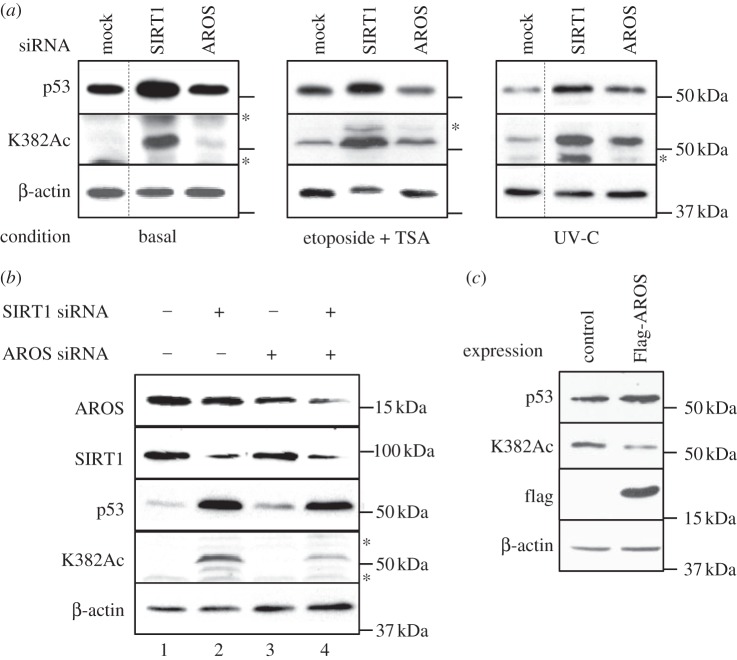
AROS does not suppress p53 acetylation under all conditions. (*a*) Western blotting for total (p53) and acetylated p53 (K382Ac) 48 h after RNAi against *SIRT1* and *AROS* in the absence of applied stress (left panel). Western blotting following stress induction by treatment with etoposide (20 µM) and trichostatin A (0.5 µM) for 6 h (middle panel) or ultraviolet (UV-C) irradiation at 10 J m^−2^ 24 h before harvesting (right panel). β-actin is used as a loading control. (*b*) Western blotting for total and acetylated (K382Ac) p53 following single or co-knockdown of AROS and SIRT1 under basal conditions. Lane 1 represents mock transfection. β-actin is used as a loading control. (*c*) Western blotting for total and acetylated p53 levels following exogenous expression of Flag-AROS in the absence of applied stress. Equal loading was assessed using β-actin. Asterisk indicates non-specific bands. Dashed lines within blots indicate removal of lanes from original autoradiographs. All experiments were performed in HCT116 cells.

The role of SIRT1 in promoting cancer cell survival has been linked to the suppression of p53 [27,28,50]. We therefore asked whether AROS suppresses p53 in HCT116 cancer cells, by monitoring total and acetylated p53 levels by western blot following RNAi against AROS, and SIRT1 for comparison. Suppression of SIRT1 results in an induction of both total and acetylated p53 (figure 5*a,* left panel). This is due to disruption of a constitutive cycle of acetylation and deacetylation of p53 at lysine residue 382 via loss of SIRT1 deacetylation activity [50]. As a result, acetylation of p53 continues, leading to increased acetyl-p53 (K382Ac). Acetylation of p53 is essential for activation of the transcriptional programme required for tumour suppression [33], and is generally followed by accumulation of p53 protein.

Should AROS promote SIRT1 activity towards p53, we would expect a similar increase in p53 acetylation following AROS knockdown, as reported previously [42]. However, we observe that targeting of AROS does not increase acetylated or total p53 levels compared with mock treatment under normal cell growth conditions (figure 5, left panel). Thus, under these conditions, AROS does not appear to be required for p53 deacetylation, suggesting that SIRT1 is able to suppress p53 when AROS expression is reduced. It is possible that incomplete silencing of AROS allows partial function and suppression of p53. However, the extent of AROS suppression achieved through RNAi-induced cancer cell apoptosis (figure 2), suggesting that a loss of AROS function has occurred.

In the light of these differences between AROS and SIRT1, we noted that the most conclusive previous data indicating a suppressive role for AROS upon p53 were obtained where p53 had been activated by the application of stress in the form of etoposide and trichostatin A [42]. This treatment induces DNA damage, and subsequently elevates p53 levels ([51,52] and electronic supplementary material, figure S3*b*). Reproducing this treatment here results in a modest increase in p53 acetylation following AROS knockdown (figure 5, middle panel). SIRT1 silencing following drug treatment mirrors the effect of silencing SIRT1 in the absence of stress, with both total and acetylated p53 levels increased.

Given the moderate suppression of p53 by AROS following etoposide and trichostatin A treatment, we assessed a second form of p53-inducing stress, ultraviolet (UV-C) irradiation (see electronic supplementary material, figure S3*b*). A single dose of 10 J m^−2^ was applied 24 h prior to harvesting cells which had been pre-treated with siRNAs against SIRT1 or AROS 48 h prior to harvesting. Following UV-C stress, knockdown of either AROS or SIRT1 results in a large increase in both total and acetylated p53 (figure 5, right panel). The extent of p53 acetylation is greater following SIRT1 knockdown, but the correlation between SIRT1 and AROS knockdown is consistent with AROS promoting SIRT1-mediated deacetylation of p53 [42]. Silencing of either SIRT1 or AROS following etoposide and trichostatin A treatment or UV-C irradiation induced an increase in apoptosis (see electronic supplementary material, figure S3*c*).

Consistent with the findings of Kim *et al*. [42], we find that overexpression of flag-tagged AROS protein under normal cell growth conditions reduces p53 acetylation (figure 5*c*). From this, it appears that AROS has the capacity to activate SIRT1 in the absence of exogenous stress, even though RNAi against AROS does not increase p53 acetylation under basal conditions. However, it is important to contrast the loss-of-function RNAi experiments in figure 5*a* with the gain-of-function overexpression experiments in figure 5*c*. Forced overexpression of AROS reveals the functions AROS is *able* to carry out, whereas removal of AROS by RNAi indicates the functions AROS is *actually* carrying out. In this instance, it appears that AROS is able to suppress p53, but under basal conditions is not doing so. Thus, in HCT116 cells, SIRT1 can continue to deacetylate p53 in the absence of AROS under basal conditions.

### 3.6. SIRT1 and active regulator of SIRT1 have different molecular functions

All together, these data indicate different molecular roles for SIRT1 and AROS in the regulation of p53; SIRT1 deacetylates p53 under all conditions analysed, whereas AROS suppression of p53 acetylation is dependent upon cell context. We assume that the regulation of p53 by AROS occurs via SIRT1, or conversely that the lack of regulation of p53 results from a lack of activation of SIRT1.

To understand this further, we analysed p53 protein following co-knockdown of AROS and SIRT1 (figure 5*b*). Surprisingly, we found that co-knockdown reduced the acetylation of p53 compared with SIRT1 knockdown alone—lanes 2 and 4. Unexpectedly, this suggests that AROS may be able to promote p53 acetylation when SIRT1 protein levels are reduced. One possible explanation is that SIRT1 regulates AROS protein and activity, reminiscent of the functional interaction SIRT1 shares with its repressor DBC1 [53], and opposing acetyl-transferase p300 [54]. While not fully understood at present, this may add an extra layer of complexity to the relationship between SIRT1, AROS and p53 that warrants further investigation.

It is important to note that the reduction in p53 acetylation following co-knockdown of AROS and SIRT1 compared with SIRT1 alone does not correlate with a reduction in apoptosis; no difference in the level of apoptosis is seen between the two conditions (see electronic supplementary material, figure S2*a*). This, and the fact that SIRT1 and AROS suppress apoptosis in HCT116 p53^−/−^ cells (figure 2), is consistent with SIRT1 and AROS enhancing cancer cell survival by p53 independent routes. Furthermore, it suggests that AROS may regulate cell survival via regulation of alternative SIRT1 targets. Future studies should analyse AROS regulation of SIRT1 targets other than p53 in an effort to understand whether AROS acts as an on/off activator or perhaps more intricately via direction of SIRT1 towards certain substrates.

### 3.7. Physiological implications of a variable SIRT1/active regulator of SIRT1 relationship

Interestingly, our data indicate a potential mechanism by which SIRT1 responds to environment cues linked to cell damage. SIRT1 may only require AROS for suppression of p53 after cellular stress. *In vitro* analyses of SIRT1 activity lend support to this non-essential role for AROS in SIRT1 activity [46,47]. Given that AROS regulation of SIRT1 depends on direct association, we note that the SIRT1–AROS interaction has recently been described as weaker than SIRT1 interactions with many other proteins [55]. These interactions were quantified from cells under basal conditions, with the potential for changes in the SIRT1 interactome following stress not analysed. Future studies will aim to elucidate the interaction and regulatory dynamics between SIRT1 and AROS.

It is possible that the variable role for AROS in p53 suppression represents relative substrate availability; following stress, the abundance of p53 is increased, perhaps rendering SIRT1 more dependent upon AROS for p53 deacetylation (see electronic supplementary material, figure S3*b*). However, p53 appears to be induced to a greater extent by etoposide and trichostatin A treatment than UV-C irradiation, yet regulation by AROS is greater following UV-C irradiation (figure 5 and the electronic supplementary material, figure S3*b*). The intra-cellular response to these insults will differ, which could account for the differing severity of the de-repression of p53 following AROS silencing. UV-C irradiation damages DNA, RNA and protein constituents of the cell [56], whereas the application of etoposide and trichostatin A will be restricted to DNA damage [51,52]. We recently identified AROS as a ribosome associated protein, important for maintaining maximal ribosome function [49]. Interestingly, the ribosome acts a hub for both RNA and protein quality control within the cell [57,58], provoking the possibility that the differences in regulation of SIRT1 by AROS are linked to regulation of AROS via its ribosomal association.

Abnormal ribosome biogenesis has long been discussed as a marker for cancer, presumably owing to a need for increased protein synthesis [59,60]. Targeting of ribosome biogenesis could provide a means to target rapidly dividing cells without mutagenic chemotherapeutics. Recently, a proof-of-concept study analysed suppression of ribosome biogenesis for anti-cancer therapy, showing excellent specificity against lymphoma cells *in vivo* [61]. Interestingly, cancer cell death was not dependent on reduced protein synthesis, but instead required p53 activation following nucleolar disruption. We have shown here that AROS is able to suppress p53 in cells exposed to stress, is required for cancer cell survival, and previously, that AROS is required for ribosome biogenesis [49]. It remains to be seen how these functions of AROS relate to each other, and to cancer cell survival.

## 4. Conclusion

We have demonstrated a cancer-specific role for AROS in the regulation of survival in a panel of human cell lines. The data suggest that AROS, as well as SIRT1, promotes survival in cancer cells while being redundant for viability in non-cancer cells. However, at the molecular level, the roles of SIRT1 and AROS differ with respect to regulation of p53. We find evidence supporting a suppressive role for AROS in regulation of p53, as previously reported [42], but also that AROS function can be suppressed with no effect on p53—which is the case under basal conditions. This indicates that SIRT1 does not require AROS as a physiological activator under all circumstances and leads to the conclusion that the positive role of AROS in regulating SIRT1 can respond to stimuli. As well as the variable suppression of p53 by AROS, this could have implications in the regulation of further SIRT1 targets, which may be regulated in a similar manner. It will be interesting to assess whether AROS is able to regulate multiple SIRT1 targets differently, suggesting that AROS has the capacity to act as a stimulus responsive orchestrator of SIRT1 activity. With SIRT1 implicated in diseases such as cancer, diabetes and neurodegeneration, greater understanding of its endogenous regulation could also lead to opportunities for therapeutic intervention.

## 5. Material and methods

### 5.1. Cell culture and treatments

All cell lines were grown at 37°C in a humidified atmosphere supplemented with 5% CO_2_. HCT116 and HCT116 p53^−/−^ epithelial colorectal adenocarcinoma cell lines were kindly provided by Prof. Bert Vogelstein [62]. These and the MCF7 (breast epithelium—ATCC HTB22) cell line are of cancerous origin. The ARPE19 (retinal pigmented epithelium—ATCC CRL-2302 [63]), WI-38 (lung fibroblast—ATCC CCL-75) and MCF10A (breast epithelium—ATCC CRL-10317) cell lines are not of cancerous origin. Etoposide and trichostatin A (Sigma) were used at 20 and 0.5 µM, respectively, for 6 h prior to harvesting of cells [42]. UV-C exposure was at a fluency of 2 J m^2^ s^−1^ with a total exposure of 10 J m^2^ as previously described [64]. SiRNA transfection and sequences for lamin AC and SIRT1 were previously described [50]. AROS siRNA sense sequences: (i) 5′-CCGUGUUCACCGAGGAAGA-(dTdT)-3′ and (ii) 5′-GACCACCUCAGAGUAAACC-(dTdT)-3′. LDH-B siRNA sense sequence: 5′-ACUUAAUCCAAUAGCCCAG-(dTdT)-3′. SiRNAs were provided by Dharmacon and applied at between 100 and 200 nM using Oligofectamine (Invitrogen). Mock cells were transfected with Oligofectamine alone. Flag-tagged AROS [42] was transfected using lipofectamine reagent and cells grown for 24 h (Invitrogen).

### 5.2. Whole cell analysis

Representative cell phenotypes were recorded using phase contrast microscopy (Olympus). Annexin V positive and propidium iodide negative staining identified early apoptotic cells by flow cytometry (Roche, Beckton–Dickinson). The percentage of apoptotic cells were adjusted relative to mock transfection set to 1. *n* ≥ 2 and error bars represent standard deviation. Degrees of significance were calculated using paired two-tailed Student's *t*-tests. Data are representative of three biological replicates.

### 5.3. Quantification of mRNA

Total RNA was purified by the RNeasy protocol (Qiagen) and used in qRT-PCR with primers for *Lamin AC* and *SIRT1* previously published [50]. *AROS* forward primer: 5′-GGAAGACGAAGGCAATTCAGGC-3′ and reverse primer: 5′-TCCTCGGTGAACACGGTGCC-3′. *n* ≥ 3 and error bars represent standard deviation. *P*-values were calculated using paired two-tailed Student's *t*-tests.

### 5.4. SDS–PAGE and immunoblotting

Cells were lysed (10 mM Tris at pH 8.0, 140 mM NaCl, 2 mM CaCl_2_, 0.5% v/v NP-40, 1× protease inhibitor cocktail (Roche) and 5 U ml^−1^ micrococcal nuclease), and the protein quantity assayed by the Pierce bicinchoninic acid method. Lysates were denatured in Laemmli's sample buffer and equivalent protein by mass analysed by SDS–PAGE. Nitrocellulose membranes were pre-blocked for 1 h then incubated in primary antibody overnight at 4°C. Antibodies for western blotting were provided by Santa Cruz (SIRT1, p53, Lamin AC), Alexis (AROS), Epitomics (acetyl-p53 K382), Sigma (Flag) and Millipore (β-actin) and were detected by HRP-conjugated secondary antibody (Dako) and POD reagent (Roche). Densitometry was carried out using the open access ImageJ software (National Institute of Health).

## Supplementary Material

Supplemental Table 1; Supplemental Figure 1: Apoptosis following co-knockdown of AROS and SIRT1 and transfection of an independent AROS siRNA; Supplemental Figure 2: AROS knockdown does not affect SIRT1 mRNA abundance; Supplemental Figure 3: SIRT1 and AROS suppress apoptosis following stress
