# Supplementary material for: Active regulator of SIRT1 is required for cancer cell survival but not for SIRT1 activity
Source: Open Biol. 2013 Nov;3(11):130130. doi: 10.1098/rsob.130130 (PMC3843821; doi:10.1098/rsob.130130)
Supplement: Supplemental Table 1; Supplemental Figure 1: Apoptosis following co-knockdown of AROS and SIRT1 and transfection of an independent AROS siRNA; Supplemental Figure 2: AROS knockdown does not affect SIRT1 mRNA abundance; Supplemental Figure 3: SIRT1 and AROS suppress apoptosis following stress [file rsob130130supp1.pptx]

## Slide 1
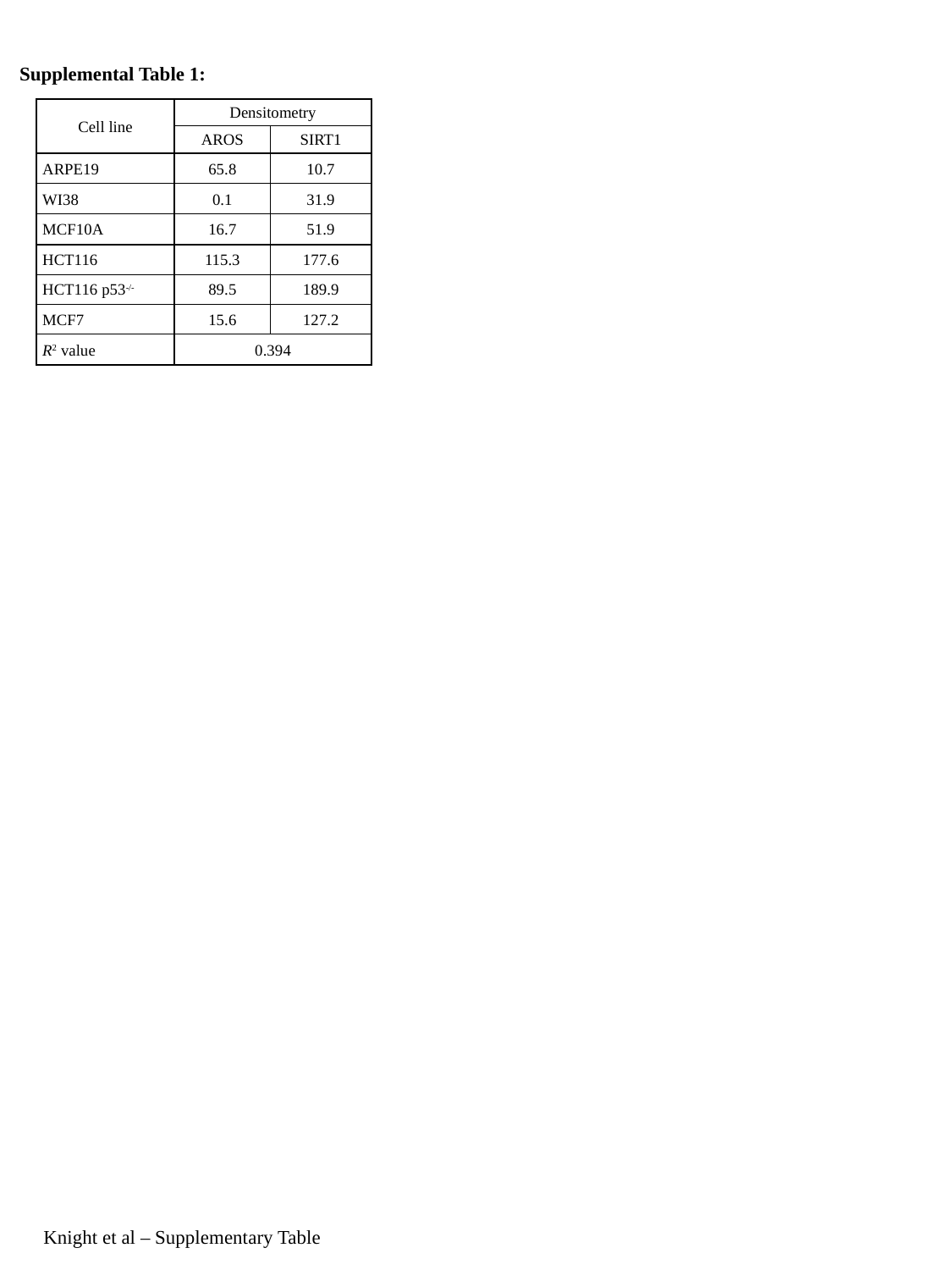

Supplemental Table 1:
| Cell line | Densitometry | |
| --- | --- | --- |
| | AROS | SIRT1 |
| ARPE19 | 65.8 | 10.7 |
| WI38 | 0.1 | 31.9 |
| MCF10A | 16.7 | 51.9 |
| HCT116 | 115.3 | 177.6 |
| HCT116 p53-/- | 89.5 | 189.9 |
| MCF7 | 15.6 | 127.2 |
| R2 value | 0.394 | |
Knight et al – Supplementary Table

## Slide 2
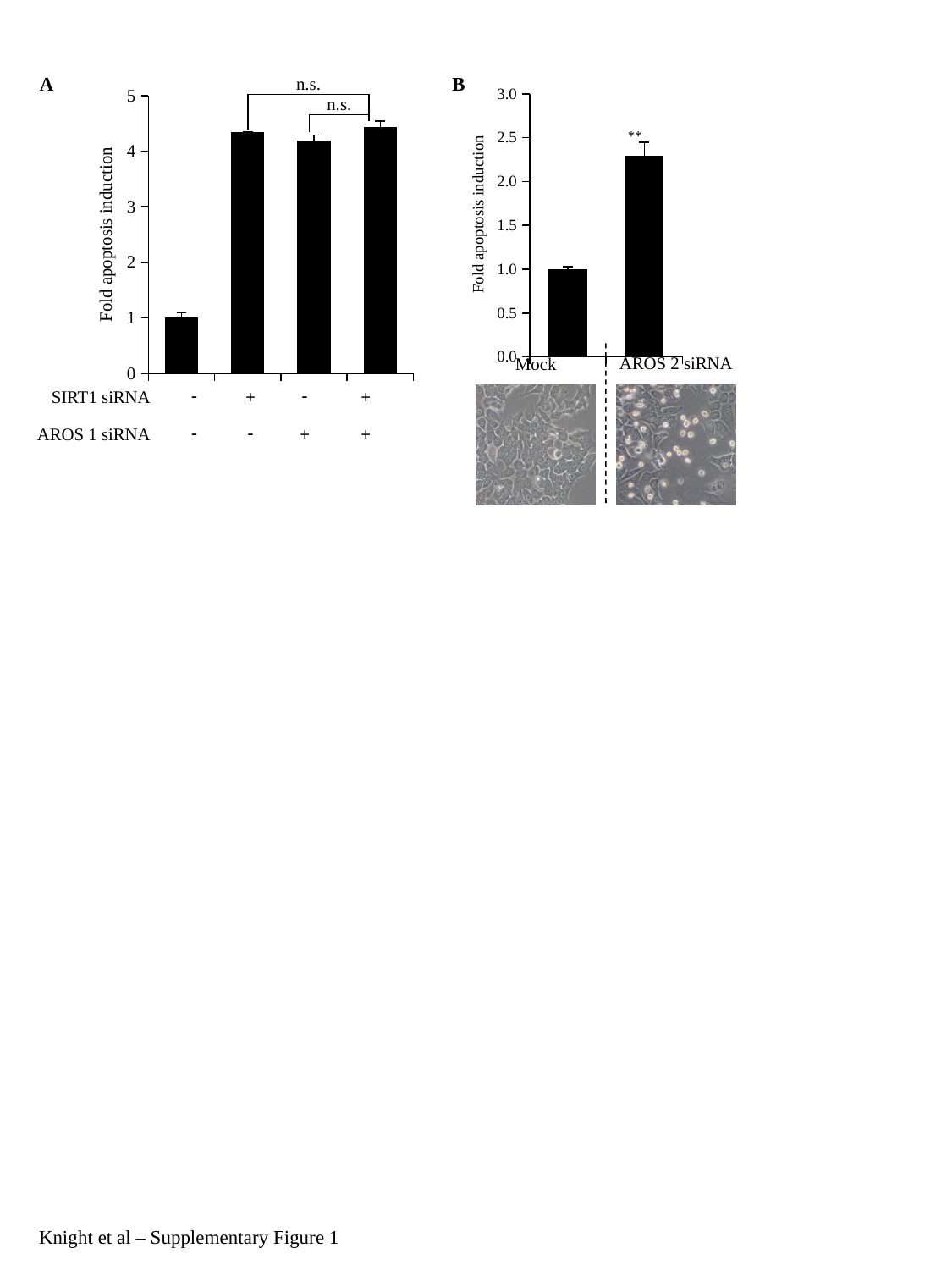

A
B
n.s.
### Chart
| Category | |
|---|---|
| Mock | 1.0 |
| AROS 2 | 2.29 |
### Chart
| Category | |
|---|---|Fold apoptosis induction
-
-
+
+
SIRT1 siRNA
-
-
AROS 1 siRNA
+
+
n.s.
**
Fold apoptosis induction
AROS 2 siRNA
Mock
Knight et al – Supplementary Figure 1

## Slide 3
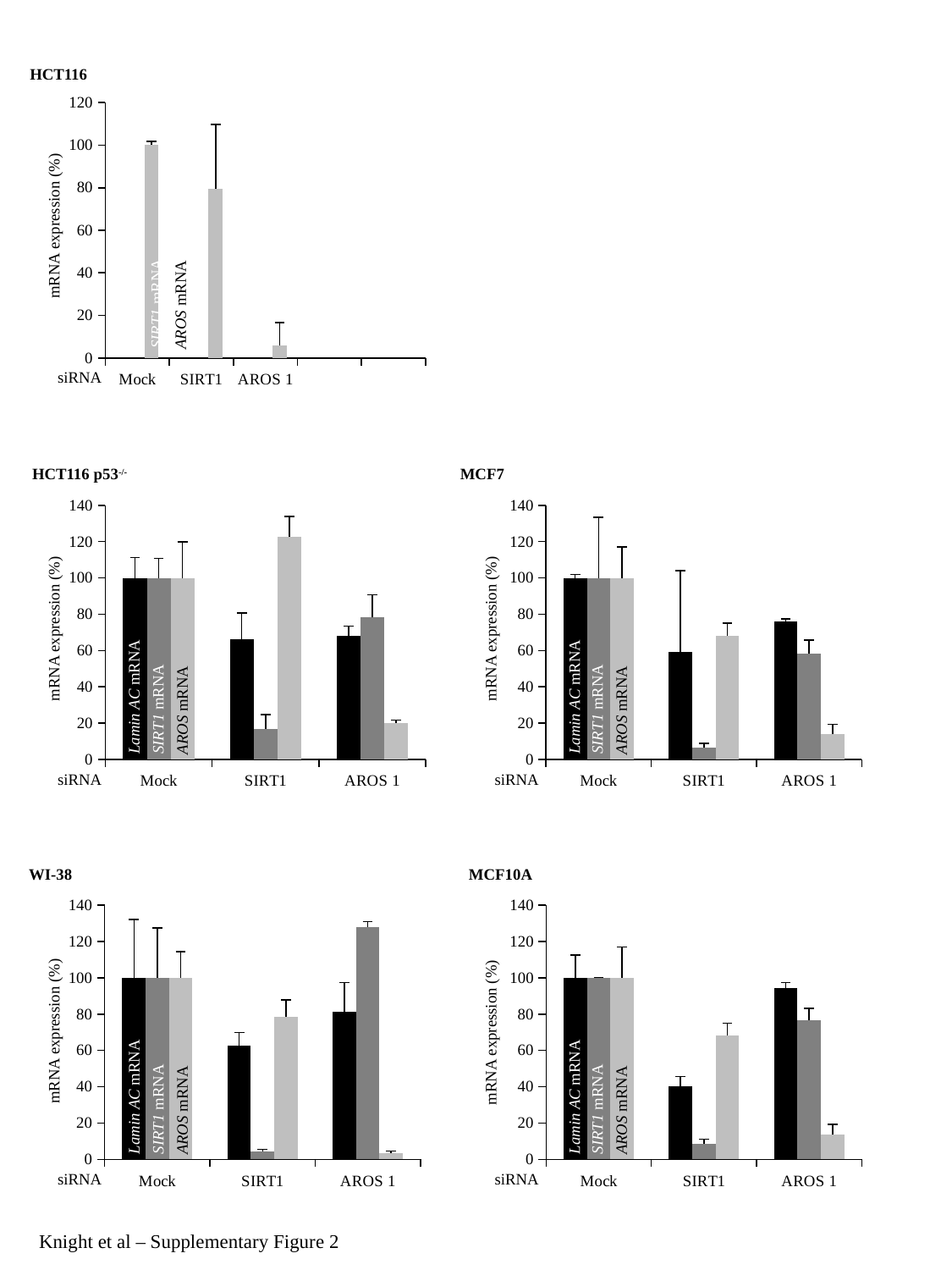

HCT116
### Chart
| Category | Lamin AC | SIRT1 | AROS |
|---|---|---|---|
| Mock | 100.0 | 100.0 | 100.0 |
| SIRT1 | 87.71186440677965 | 10.60958059210526 | 79.68207564407088 |
| AROS 1 | 107.76836158192091 | 72.6014254385965 | 5.740909921432486 | mRNA expression (%)
SIRT1 mRNA
Lamin AC mRNA
AROS mRNA
siRNA
HCT116 p53-/-
MCF7
### Chart
| Category | Lamin AC | SIRT1 | AROS |
|---|---|---|---|
| Mock | 100.0 | 100.0 | 100.0 |
| SIRT1 | 66.17839556192732 | 16.8041166380789 | 122.43298969072166 |
| AROS 1 | 68.03798125870057 | 78.16809605488851 | 20.120962199312714 |
### Chart
| Category | Lamin A/C | SIRT1 | AROS |
|---|---|---|---|
| Mock | 100.0 | 100.0 | 100.0 |
| SIRT1 | 59.03439951719975 | 6.739063272367771 | 68.28072494904424 |
| AROS 1 | 75.90826795413398 | 58.22417202174988 | 13.87098551203658 | mRNA expression (%)
 mRNA expression (%)
SIRT1 mRNA
SIRT1 mRNA
Lamin AC mRNA
Lamin AC mRNA
AROS mRNA
AROS mRNA
siRNA
siRNA
WI-38
MCF10A
### Chart
| Category | Actin | SIRT1 | AROS |
|---|---|---|---|
| Mock | 100.0 | 100.0 | 100.0 |
| SIRT1 | 62.44081059390049 | 4.49057443966157 | 78.49606182196462 |
| AROS 1 | 81.22993579454254 | 128.06887338578002 | 3.610789121711992 |
### Chart
| Category | Lamin A/C | SIRT1 | AROS |
|---|---|---|---|
| Mock | 100.0 | 100.0 | 100.0 |
| SIRT1 | 40.316637095673535 | 8.349793293523197 | 68.28072494904424 |
| AROS 1 | 94.33601746963286 | 76.83739090491501 | 13.87098551203658 | mRNA expression (%)
 mRNA expression (%)
SIRT1 mRNA
SIRT1 mRNA
Lamin AC mRNA
Lamin AC mRNA
AROS mRNA
AROS mRNA
siRNA
siRNA
Knight et al – Supplementary Figure 2

## Slide 4
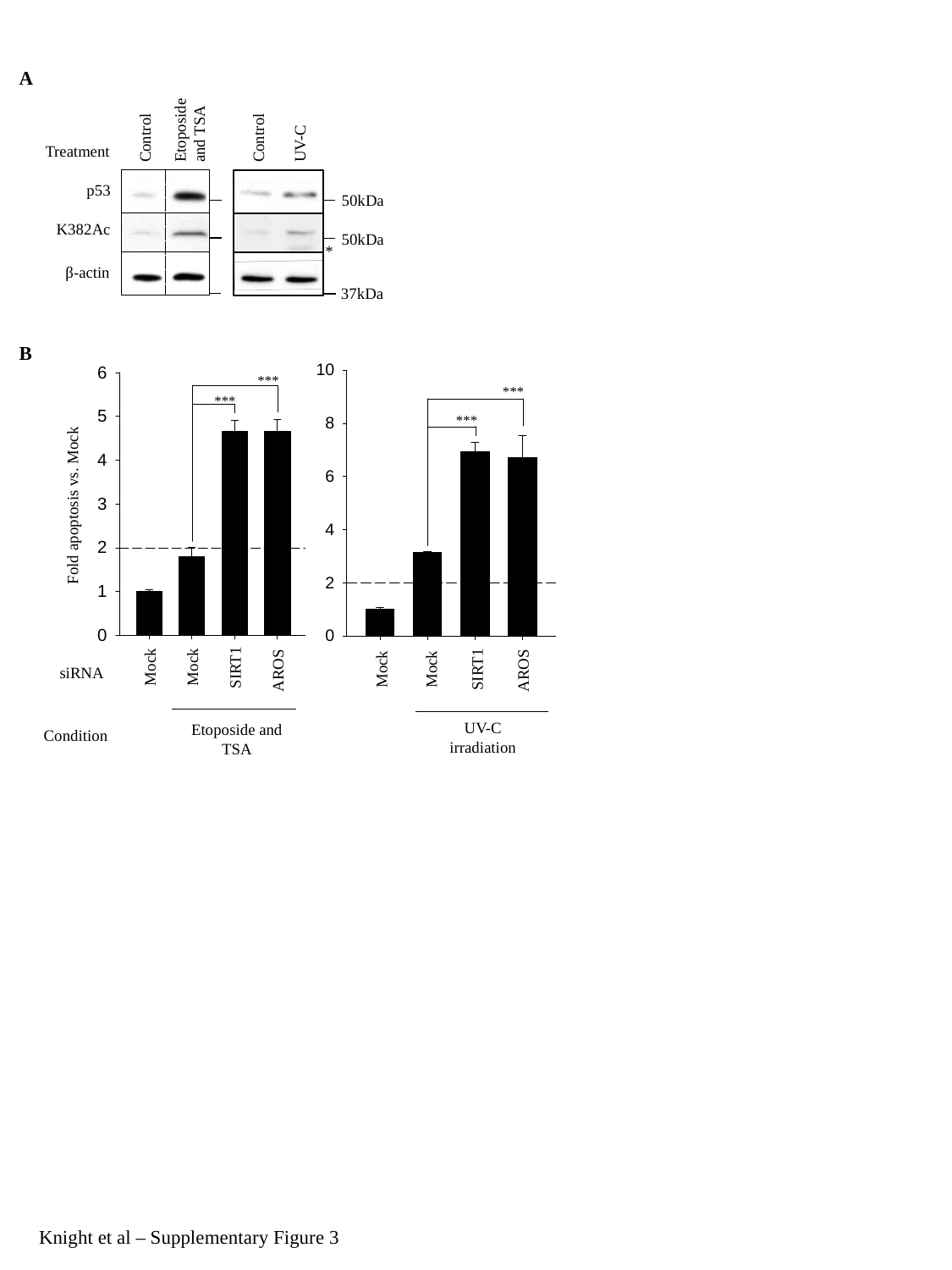

A
Etoposide
and TSA
Control
Control
UV-C
Treatment
p53
50kDa
K382Ac
50kDa
*
β-actin
37kDa
B
***
***
***
***
Fold apoptosis vs. Mock
Mock
Mock
SIRT1
Mock
Mock
SIRT1
AROS
AROS
siRNA
UV-C irradiation
Etoposide and TSA
Condition
Knight et al – Supplementary Figure 3
